# Protein language models learn evolutionary statistics of interacting sequence motifs

**DOI:** 10.1073/pnas.2406285121

**Published:** 2024-10-28

**Authors:** Zhidian Zhang, Hannah K. Wayment-Steele, Garyk Brixi, Haobo Wang, Dorothee Kern, Sergey Ovchinnikov

**Affiliations:** ^a^Harvard University, Cambridge, MA 02138; ^b^Department of Biology, Massachusetts Institute of Technology, Cambridge, MA 02139; ^c^Institute of Bioengineering, School of Life Sciences, Ecole polytechnique fédérale de Lausanne, Lausanne VD 1015, Switzerland; ^d^HHMI, Brandeis University, Waltham, MA 02453; ^e^Department of Biochemistry, Brandeis University, Waltham, MA 02453; ^f^Harvard College, Harvard University, Cambridge, MA 02138; ^g^John Harvard Distinguished Science Fellowship, Harvard University, Cambridge, MA 02138

**Keywords:** language models, interpretability study, protein structure prediction

## Abstract

Protein language models (pLMs) have emerged as potent tools for predicting and designing protein structure and function, and the degree to which these models fundamentally understand the inherent biophysics of protein structure stands as an open question. Motivated by a finding that pLM-based structure predictors erroneously predict nonphysical structures for protein isoforms, we investigated the nature of sequence context needed for contact predictions in the pLM Evolutionary Scale Modeling (ESM-2). We demonstrate by use of a “categorical Jacobian” calculation that ESM-2 stores statistics of coevolving residues, analogously to simpler modeling approaches like Markov Random Fields and Multivariate Gaussian models. We further investigated how ESM-2 “stores” information needed to predict contacts by comparing sequence masking strategies, and found that providing local windows of sequence information allowed ESM-2 to best recover predicted contacts. This suggests that pLMs predict contacts by storing motifs of pairwise contacts. Our investigation highlights the limitations of current pLMs and underscores the importance of understanding the underlying mechanisms of these models.

## Significance

Protein language models (pLMs) have exhibited remarkable capabilities in protein structure prediction and design. However, the extent to which they comprehend the intrinsic biophysics of protein structures remains uncertain. We present a suite of analyses that dissect how the flagship pLM ESM-2 predicts structure. Motivated by a consistent error of protein isoforms predicted as structured fragments, we developed a completely unsupervised method to uniformly evaluate any pLM, allowing us to compare coevolutionary statistics to linear models. We further identified that ESM-2 does not require full context for predicting interresidue contacts. Our study highlights the current limitations of pLMs and contributes to a deeper understanding of their underlying mechanisms, paving the way for more reliable protein structure predictions.

Determining the structure of a protein is a critical first step to understanding its function in biology; therefore, tremendous efforts have been devoted to the task of predicting protein structure from sequence. AlphaFold2 (AF2) (1) dramatically improved the prediction accuracy of single protein structures in the Critical Assessment of protein Structure Prediction (CASP14) challenge. Central to AF2’s methodology are multiple sequence alignments (MSA) that contain information on evolutionary couplings between amino acids within a structure. However, proteins’ folding in solution know nothing of their evolutionarily related counterparts and methods that can accurately predict structure from a single sequence alone would ideally bring us closer to understanding the biophysics of protein folding. Furthermore, using MSAs to predict structure limits the usefulness of these methods in contexts where few sequence homologs are available. These motivations have driven the development of single-sequence, i.e. MSA-free, structure prediction methods, such as OmegaFold (2), Recurrent Geometric Network (RGN2) (3), and ESMFold (4). OmegaFold is based on the protein language model OmegaPLM, RGN2 is based on the language model aminoBidirectional Encoder Representations from Transformers (aminoBERT), and ESMFold is based on the protein language model Evolutionary Scale Modeling (ESM-2). Given that these methods do not require MSAs as input, this has raised the question whether protein language models have learned the intrinsic physics of folding a single amino acid sequence? More generally, how do they achieve high predictive accuracy from a single sequence? A deeper understanding and interpretation of these models is needed for them to be used reliably. We speculated that though superficially, MSA-based methods such as AF2 and protein language models may appear quite different in their input information (MSA vs. unaligned sequences) and training (supervised on structure vs. unsupervised), the two methods may be achieving the same outcome, be it explicitly learning to extract the coevolutionary information from input MSA or implicitly learning to lookup the same stored evolutionary information in the parameters of the model.

**Fig. 1.**
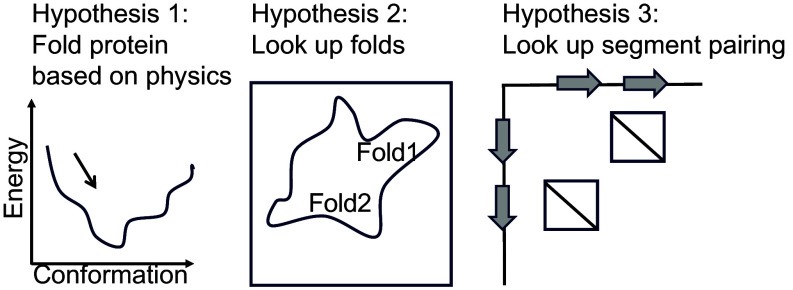
Three hypotheses of how language models predict protein structures.

In this work, we dissected how the language model ESM-2 enables highly accurate structure prediction by evaluating three different hypotheses for its function (Fig. 1). We start with hypothesis 1 that ESM-2 truly has learned protein folding from physics. This is already contradicted by the result that ESM-2 performance is highly correlated with the number of sequence neighbors in the training set across all model sizes (4, 5). If ESM-2 truly had learned the physics of protein folding, its performance should not depend on the number of sequence neighbors of a given protein. This hypothesis was further contradicted by a striking consistent error we observed in structure predictions for isoforms from alternative splicing—some of which, from the perspective of a sequence-based model, can be thought of as fragments of full-length sequences. Based on this finding, we formulated two alternate hypotheses. Hypothesis 2 is that ESM-2 stores a separate coevolution model for each protein family (at the domain or fold level). Given an entire protein sequence, it would match contact predictions to a particular protein family. Alternatively, hypothesis 3 is the model stored small coevolutionary models for each pair of interacting fragments that are independent of each other and may be shared across protein families. We designed a series of experiments to test these hypotheses and provide evidence for supporting the third hypothesis: that ESM-2 has learned pairwise dependencies conditioned on sequence motifs and the relative separation between the sequences. This suggests an analogous mechanism to many prior approaches to predict and design protein structure using modular sets of interacting motifs (6–9).

## Results

### Language Models Predict Unrealistic Structures for Protein Isoforms.

Protein isoforms are proteins that originate from a single gene family and formed from alternative splicings or other posttranscriptional modifications (10). Protein isoforms resulting from splicing events within structured domains have long been presented as a pathology for homology-based structure modeling (11–14), since their sequences are very similar to their full-length proteins, yet are often likely unfolded and nonfunctional (14). These isoforms offered an opportunity to evaluate the capabilities of the current protein structure prediction methods. If state-of-the-art protein structure prediction approaches predict such isoforms as either unfolded or alternately structured, it would imply an intrinsic understanding of the biophysics of protein folding. We curated a dataset of 18 domain-splitting isoforms that had previously been identified in refs. 11–14, and made structure predictions using AlphaFold2 (with MSA input), OmegaFold (language model), and ESMFold (language model) (*Materials and Methods*) (15).

**Fig. 2.**
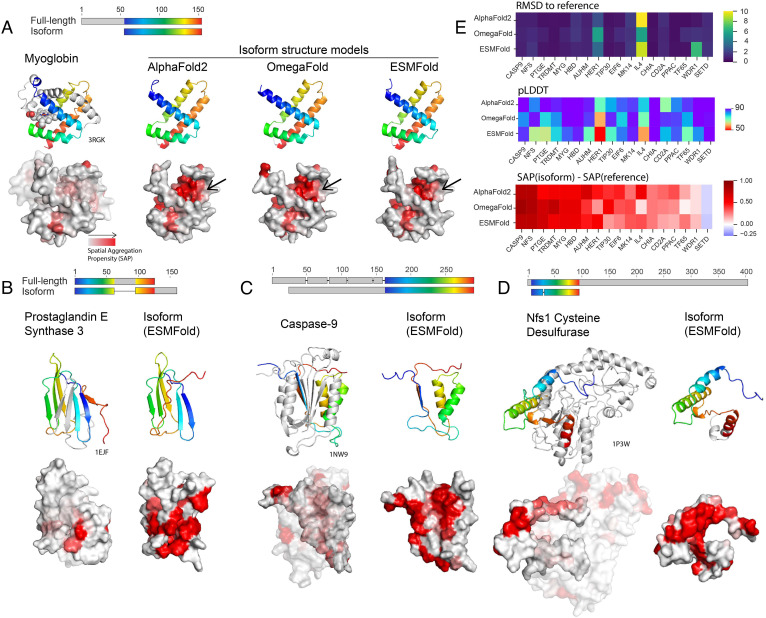
Deep learning structure-based methods predict isoforms as fragments of full-length structures with exposed aggregation-prone residues. (*A*) *Top*: alignment between human myoglobin (UniProt: P02144) and human isoform Q8WVH6 (UniProt: Q8WVH6). *Left*: X-ray structure of human myoglobin [Protein Data Bank (PDB): 3RGK] shown with the heme cofactor, and the two missing helices A and B in the isoform colored in gray. *Right*: predicted structures of isoform Q8WVH6 of Myoglobin from AlphaFold2, OmegaFold, and ESMFold have low root-mean-squared deviation (RMSD) to structure 3RGK (0.49, 1.01, and 0.81 Å respectively). *Bottom*: surfaces of protein fragments corresponding to the isoform sequence. The isoform structure models all have exposed hydrophobic residues corresponding to where helices A and B reside in the full-length structure (indicated with an arrow), quantified here using the spatial aggregation propensity (SAP) score (16). Structure predictions of isoforms from Human Prostaglandin E synthase 3 (*B*), Human Caspase-9 (*C*), and Human Nfs1 cysteine desulfurase (*D*) all share similar trends, where the isoform structure model contains a significant patch of residues with high SAP score. In (*B*–*D*), the structure model depicted is from ESMFold. (*E*) For 18 isoforms previously identified in the literature as isoforms where splicing events occur in structured domains, we calculated RMSD to a reference structure of the full-length protein, and the change in average SAP for the isoform fragment in comparison to the sequence aligned in the full-length protein. We found that for AlphaFold2, OmegaFold, and ESMFold, isoform structure models generally had low RMSD to the reference structure, predicted with relatively high pLDDT, along with increased average SAP.

An example isoform from human myoglobin, first discussed as an example of this phenomenon in ref. 13, is depicted in Fig. 2*A*. The isoform’s predicted structures in AF2, OmegaFold, and ESMFold have 0.49, 1.01, and 0.81 Å root-mean-squared deviation (RMSD), respectively, to the segment of the full-length protein that aligns to the isoform. However, this three-dimensional fold is improbable: Multiple hydrophobic residues are exposed in a cleft that in the full-length form of myoglobin, would be occupied by helices A and B. We quantified this effect using the spatial aggregation propensity (SAP) score (16). The *Bottom* row of Fig. 2*A* depicts the surface of the sequence corresponding to the isoform within the full-length protein, as well as the isoform structure models, colored by the calculated per-residue SAP score. Structure predictions of isoforms from human Prostaglandin E synthase 3 (Fig. 2*B*), human Caspase-9 (Fig. 2*C*), and human Nfs1 cysteine desulfurase (Fig. 2*D*) all share similar trends, where the isoform structure model contains a significant patch of residues with high SAP score. We observed low RMSD to the reference full-length structure, accompanied by high model confidence and increased mean SAP scores across many isoforms (Fig. 2*E*), indicating both MSA-based and Protein language models, pLM-based models are prone to the error of predicting structures of modified sequences within the context of the full-length protein, countering hypothesis 1.

### An Unsupervised Method of Extracting Coevolutionary Signal from Language Models.

**Fig. 3.**
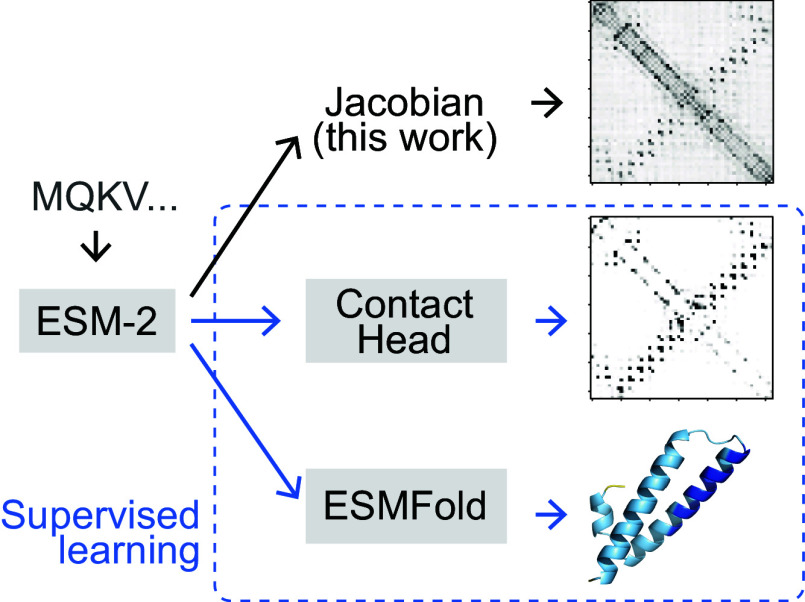
Scheme comparing strategies to extract structure and coevolutionary information from the language model ESM-2. We present an unsupervised “categorical Jacobian” calculation to extract coevolutionary couplings.

Following our observations regarding isoforms, we proceeded to further explore how the language model ESM-2, the language model underlying ESMFold (Fig. 3), predicts contacts and how it might be storing coevolutionary information. In ref. 17, the authors first developed a method for contact prediction by supervising training on attention matrices from within the language model, the so-called “Contact Head.” Ref. 4 furthered this work by developing the “Folding Trunk” to predict 3D structure from ESM-2 embeddings. Both of these extensions to the original ESM-2 model were developed using supervised learning on sets of contacts or 3D structures. We wished to develop an approach to evaluate coevolutionary signal in a completely unsupervised manner, to understand what information the original ESM-2 model, trained only using the unsupervised task of masked language modeling, holds. We formulated the “categorical Jacobian” calculation (Fig. 3) described below toward this end.

**Fig. 4.**
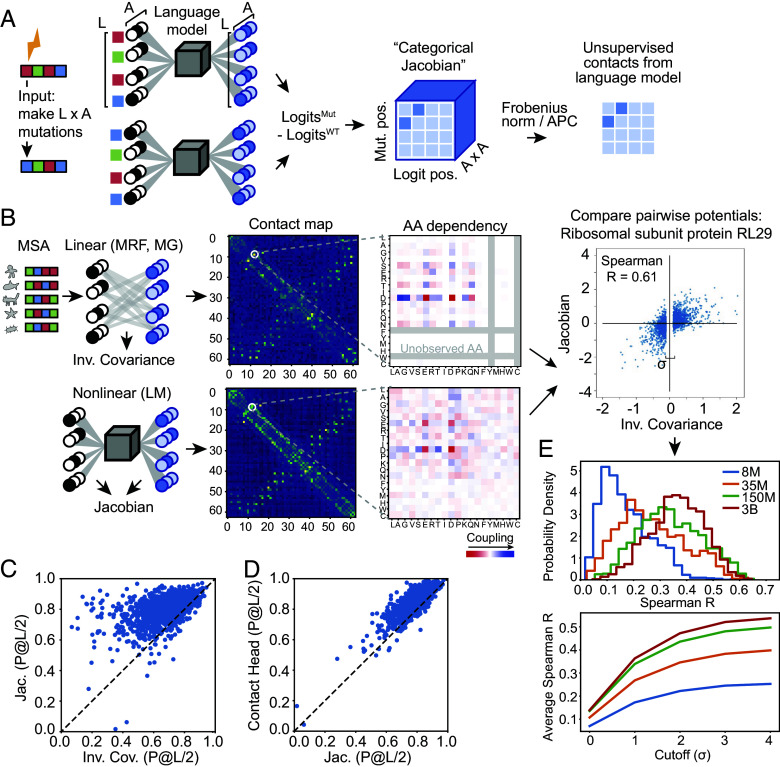
Categorical Jacobian is an unsupervised method to extract coevolutionary signal and uniformly evaluate any pLM. (*A*) Scheme of the categorical Jacobian calculation. Each residue in a sequence of length L is changed to A different types of amino acids, where A is the size of the alphabet (for proteins, A=20). By computing how the output changes with respect to the input, a matrix of size [L, A, L, A] is obtained. (*B*) This categorial Jacobian allows for comparing a nonlinear method like ESM-2 and a simple linear method, exemplified here for large ribosomal subunit protein RL29 (UniProt: P0A7M7). We can compare the coevolutionary weights obtained from a linear model, calculated using inverse covariance, and the categorical Jacobian calculated from ESM-2. (*C*) Contacts calculated from the categorical Jacobian from ESM-2 outperform the inverse covariance calculation from ref. 25 (Average long-range P@L/2 of 0.67 vs. 0.80, respectively). (*D*) Comparing contact accuracy from the categorical Jacobian and the supervised contact prediction head (Average long-range P@L/2 of 0.80 vs. 0.87, respectively). (*E*) Correlation between covariation parameters from linear model and ESM-2 Jacobians increase with model size. *Top*: Distribution of Spearman correlation coefficients between contacts from linear model and ESM-2 Jacobians. *Bottom*: average Spearman R, varying σ cutoff for linear model values close to zero. For (*C*–*E*), N = 1,431 proteins.

**Fig. 5.**
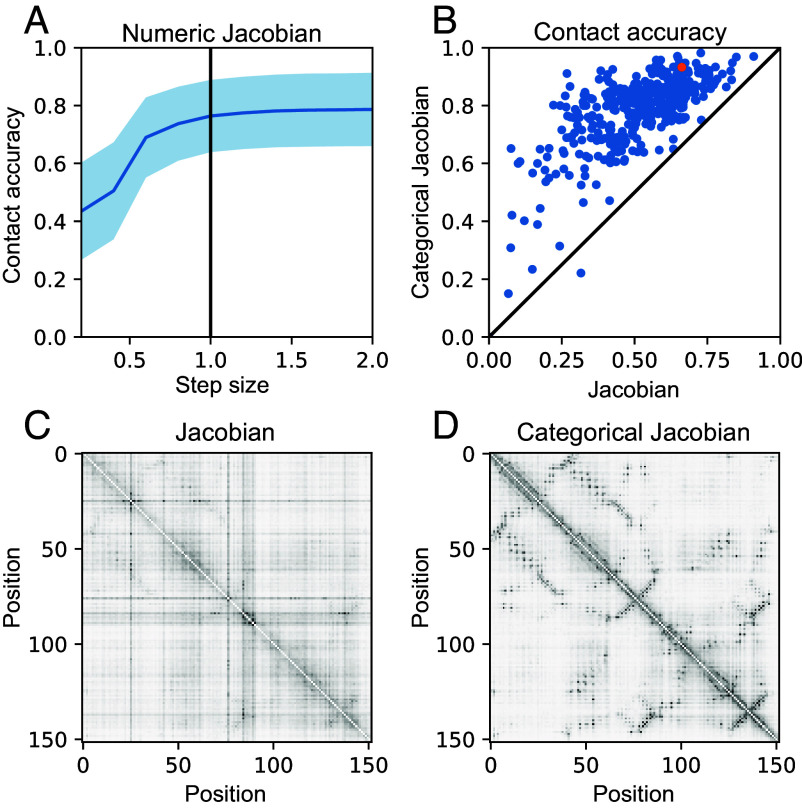
Categorical Jacobian has higher contact accuracy. (*A*) Jacobian derived via numeric differentiation shows a large step size is required to obtain the best accuracy. (*B*) Comparing Jacobian to Categorical Jacobian across a set of 383 proteins (*SI Appendix*, *Methods*). The orange dot highlights example PDB: 3A0Y which is the Catalytic domain of histidine kinase ThkA. (*C*) Jacobian of PDB: 3A0Y. (*D*) Categorical Jacobian of PDB: 3A0Y.

For a biological sequence of length L with A possible tokens (i.e., amino acids for proteins), we extract a set of weights defining the “categorical Jacobian” J as follows (illustrated in Fig. 4*A*). We mutate each residue in the sequence to each of A possible tokens, and calculate how each of these L×A mutations perturbs the probabilities of each amino acid across all positions output by the language model, i.e. the logits, which have shape L×A. Accordingly, the shape of the tensor J is L×A×L×A. Applying the same procedure to a Markov Random Field (MRF) (18–20) or multivariate Gaussian (MG) (21) model results in exactly returning the pairwise coupling tensor $W^{L,A,L,A}$, and could be also calculated by perturbing the value of the original token, yet we found that in the context of ESM-2, this “categorical” perturbation is critical. In a linear model (MRF or MG), perturbation of any step size returns the same value in the Jacobian (22), yet in ESM-2, a small perturbation to the one-hot encoded input is insufficient to perturb the output (Fig. 5*A*–*C*). We noticed that increasing the step size improves contact map accuracy (Fig. 5*A*) and changing the actual category (amino acid type) results in the best contact accuracy (Fig. 5*D*). This unsupervised Jacobian method allows us to directly compare pairwise coupling weights from language models to pairwise coupling weights derived from MRF and MG-based models.

With this categorical Jacobian calculation in hand as an unsupervised approach for assessing pairwise coevolutionary weights of pLMs, we next set out to evaluate how these pairwise weights compare to linear models in the task of contact prediction, as well as the supervised “Contact Head” that was trained on top of ESM-2 embeddings. From our Jacobian tensor, we can calculate a predicted contact map of size L×L analogously to MRFs and MGs (*Materials and Methods*) (23, 24). Fig. 4*B* depicts an example comparison of pairwise coevolutionary weights for the large ribosomal subunit protein RL29 calculated with 2 methods: on the *Top*, using a multivariate Gaussian approach inferred from an MSA for the family (25), and on the *Bottom*, from the categorical Jacobian of ESM-2 with 3 billion parameters. The *Left* coloumn depicts summed contact weights from both methods and the *Right* column depicts an example 20 × 20 set of weights corresponding to pairwise amino acid dependencies for a given pairwise contact, demonstrating striking visual similarities between the two methods. Analogous sets of weights for other ESM-2 model sizes are depicted in *SI Appendix*, Fig. S1. We note that in the couplings calculated from a set number of sequences in an MSA, some residue types may not be observed in every position, and the couplings, therefore, cannot be inferred (indicated in grey in Fig. 4*B*). In contrast, pLM infers a pairwise coupling value for every residue type at every position, including interactions which may never appear in the finite number of sequences in the MSA.

We compared the accuracy of contacts predicted with a standard linear model for pairwise couplings (25, 26) or predicted with the categorical Jacobian of the ESM-2 3-billion-parameter model, quantifying accuracy via precision of the *L/2* top-weighted long-range contacts (*Materials and Methods*). We used the 3-billion-parameter model because it showed similar performance to the 15-billion-parameter model (4). The categorical Jacobian calculation demonstrated improved performance at predicting contacts than the linear model across our dataset of 1,431 proteins (see *Materials and Methods* for dataset construction) (Fig. 4*C*, average accuracy of 0.80 and 0.67, respectively). Contacts from the categorical Jacobian had lower accuracy than the supervised contact head (Fig. 4*D*), average accuracy of 0.80 and 0.87, respectively).

Next, we were curious how similar the actual underlying weight matrices were between these two methods, i.e., a linear model and the ESM-2 categorical Jacobian calculation. Estimating a linear model involves fitting L×A×L×A parameters for each family, which is very likely overdetermined, and many of the weights are driven to zero. We assessed the correlation at different cutoffs of removing weights closest to zero (*Materials and Methods*). In our benchmark of 1,431 proteins, we found that the correlation between pairwise coupling weights from ESM-2 and from a linear model increased with the size of the ESM-2 model (Fig. 4*E*), with performance plateauing at the 150-million to 3-billion parameter model sizes.

### Language Models Predict Structures by Looking Up Segment Pairings.

**Fig. 6.**
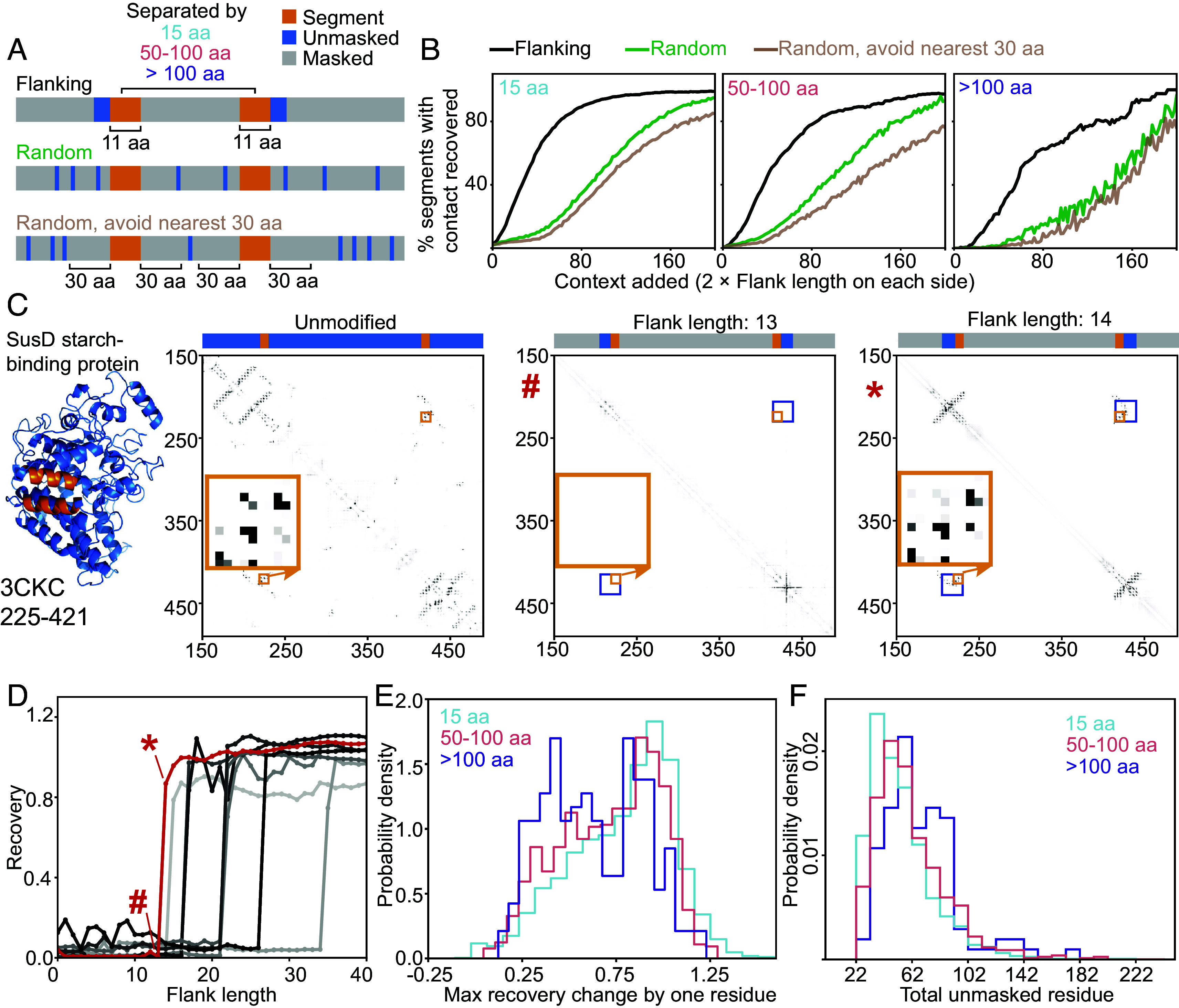
Contact recovery experiments revealed that pLMs predict structures by looking up segment pairings. (*A*) Scheme depicting segment pair contact recovery experiment. Orange: segment where contact is detected. Blue: unmasked aa. Gray: masked aa. (*B*) The percentage of segment pairs with contact recovered at different numbers of unmasked residues. N = 4,022, 1,273, and 304 segment pairs for segments separated by 15 aa (cyan), 50 to 100 aa (pink), and >100 aa (purple). (*C*) *Left*: Structure of starch-binding protein SusD with example helix–helix contact visualized in orange (PDB: 3CKC). *Right*: ESM2 contact maps of fully unmasked and partially unmasked sequences. The contact prediction is not present with 13 residues unmasked (marked with #), but appears with 14 residues unmasked (marked with *). (*D*) Contact recovery at different unmasked flank lengths for 3CKC 225-421 (red) and other segment pairs from other proteins (gray). (*E*) Distribution of the maximum recovery increased from adding one residue to the unmasked flanking region (calculated for segment pairs that reached a contact recovery of 0.5). N = 3,264, 1,027, and 170 segment pairs for segments separated by 15 aa, 50 to 100 aa, and >100 aa. (*F*) Distribution of total unmasked residues at the “jump” of recovery by asymmetrical unmasking of the outer flanking regions (calculated for segment pairs with a “jump” in contact recovery). N = 2,625, 763, and 103 segment pairs for segments separated by 15 aa, 50 to 100 aa, and >100 aa, respectively.

Given that we could calculate a Jacobian of ESM-2 that contained coevolutionary signal rivalling the information predicted by the supervised Contact Head, we wished to more thoroughly investigate the mechanism of precisely how the Contact Head predicts contacts given an input sequence. We tested what information is most used in contact prediction by monitoring prediction from the Contact Head when information from various sequence locations is masked. We used the Contact Head for these experiments because it is faster to compute than the categorical Jacobian for large-scale studies. We first masked the whole sequence and only unmasked two 11 aa segments to examine their interaction. Then, we compared three unmasking strategies to disentangle the impact of local context and global context on contact prediction. The first approach was unmasking residues flanking the target segments to reveal the impact of local context. As controls, we either randomly unmasked the same amount of residues throughout the protein or randomly unmasked residues but avoided unmasking residues within 30 aa to the termini of the segments (Fig. 6*A*). We hypothesized that a model that stores local motifs would better be able to recover contacts by gradually unmasking residues next to the contact in question while a model that stores complete domains or folds would be able to recover contacts similarly via randomly unmasking residues and unmasking flanking regions. Testing these unmasking strategies revealed that ESM-2 more rapidly recovered contacts by unmasking flanking regions, with 50% being recovered with approximately 16 residues unmasked on each side, and contact recovery from flanking unmasking being roughly 3 times as effective as randomly unmasking (Fig. 6*B* and *SI Appendix*, Table S1).

We found similar trends when analyzing how much context ESM-2 needs to recover contacts between more distant secondary structure elements (SSEs). We took two 11-residue segments from a pair of interacting SSEs, with centers separated by at least 50 residues, and masked the rest of the protein. Then, we gradually unmasked more flanking regions on the outer sides of the segments (Fig. 6*A*) and monitored the contact recovery. We found that 50% of the SSE pairs’ contacts were restored with a flanking length of 22 or 30 residues (Fig. 6*F*) for pairs separated by 50 to 100 or more than 100 residues, respectively. The contact recovery from flanking unmasking is roughly 2.5 times as effective as random unmasking (Fig. 6*B* and *SI Appendix*, Table S1).

We observed a striking step-function type behavior in how ESM-2 uncovered contacts while unmasking flanks for the starch-binding protein SusD (PDB: 3CKC) (27) (Fig. 6*C*). ESM-2 shifted from not predicting the contact between two α-helices centered at residue 225 and residue 421 using 13 flanking residues on each side to complete contact recovery at 14 residues (Fig. 6 *C* and *D*). This intriguing contact recovery pattern was observed in multiple cases (Fig. 6*D*) and motivated us to characterize the prevalence (*Materials and Methods* and *SI Appendix*, Figs. S2–S4). We calculated the maximal recovery increase achieved upon adding one residue for each segment pair (Fig. 6*E*). For segments separated by 15 aa, 50 to 100 aa, and > 100 aa, 82%, 76%, and 64% out of these that reached recovery have a “jump” of more than 0.5 in recovery with one residue. The number of total unmasked residues needed for 90% of these jumps to occur was between 85 to 94 (Fig. 6*F*), which we calculated by unmasking asymmetrically to ensure we found the more precise motif size (*Materials and Methods*). Alternate behaviors upon unmasking that did not fall into our “jump” classification are described in *SI Appendix*, Figs. S2–S4.

One limitation of our study is that we are only unmasking residues flanking the outward regions of the segment, and such unmasking could also be done inward. Thus, the minimal total unmasked residues needed for recovery might be lower than the number we showed.

Further, we observed contacts predicted even for some masked part of the sequence (Fig. 6*C* and *SI Appendix*, Fig. S3, contacts outside the blue boxes), indicating that in some cases the size of the learned motif may be larger than the amount of context used to recover part of the motif.

## Discussion

The development of pLMs has brought significant excitement into the field of protein structure prediction. Some have wondered whether pLMs have finally solved the “protein folding problem,” given their accurate structure prediction from single sequences and no supplied coevolutionary signal in an input multiple sequence alignment (2). This was quickly debunked, as the accuracy of models was found to be highly correlated to the number of related proteins in the training set (3, 4), indicating that the models store evolutionary information in their parameters, but precisely how has been unclear.

A clue for how ESM-2 might be storing coevolutionary information came via a consistent error we encountered in the predicted structures of isoforms, which we found were consistently predicted to fold to fragments matching their structure context within the full-length proteins, but which left nonphysical patches of hydrophobic residues exposed. We figured whether the model learned protein folding and not simply looked up evolutionary statistics, it should be able to model a more-likely unfolded conformation. Our results caution against assuming pLMs as oracles of protein properties without consideration of potential adversarial and out-of-distribution behaviors. Notably, AF2 is prone to this error as well. As of January 2024, we identified one such erroneous structure, Myoglobin isoform CHS.35702.2 predicted with predicted Local Distance Difference Test (pLDDT) 94.3 (*SI Appendix*, Fig. S5), from the isoforms we analyzed in the “Comprehensive Human Expressed SequenceS (CHESS) human protein structure database” public database of AF2 isoform predictions (28). A clear limitation of this study is that we do not have experimental evidence for the actual in vitro structure landscapes of these isoform examples.

Motivated to develop a framework to assess coevolutionary signals within language models, we developed a general calculation to calculate a “categorical Jacobian” of a pLM for a given sequence. The values of the categorical Jacobian can be directly compared to the pairwise weights of a Markov Random Field (18–20) or multivariate Gaussian model (21) calculated for a given MSA, approaches which have long been used to assess coevolutionary couplings in protein families.

Another approach to extract contacts in an unsupervised way from nonlinear models is to do all combinations of single and double mutations and take the differences (double—singles) in the likelihoods (29). Though this is similar, it is prohibitively expensive to compute for pLMs, and we find doing a scan of single mutations is all that is needed to extract the pairwise dependencies.

We were curious if we could detect patterns in how ESM-2 uses coevolutionary information to predict contacts. We tested unmasking residues in various patterns surrounding contacts, which revealed that the model best recovers contacts by gradually unmasking residues next to the contact in question compared to random unmasking. This suggests that pLMs learned statistics of motif pairings. We suggest that this relationship can be roughly represented as:

[1] $$\begin{array}{c} P(\;\text{contact}[a,b]|\;\text{seq}[a:a\pm s],\;\text{seq}[b:b\pm s]), \end{array}$$

where s depends on the motif the contact is in. Without this context, the pLMs are unable to correctly predict the interaction between fragments.

Our analysis does not completely rule out that pLMs have learned the concept of full folds, since the continuous segment unmasked in the flanking region might have helped the model to match to full proteins. Nevertheless, our results underscore that the information of the full fold is not required for the model to function.

Storing the coevolutionary statistics^*^ of all known protein families in UniProt (roughly 20,000), assuming an average length of 256, would require

[2] 20,000families×2562pairwise interactions×202amino acids=261billion parameters.

If we assume each position makes at most 4 contacts—2 sequence neighbors and roughly 2 long-range contacts—this corresponds to

[3] $$\begin{array}{cc} & 20,000\;\text{families}\times 256\times 4\;\text{pairwise interactions}\\ & \;\times \;20^{2}\;\text{amino acids}=4\;\text{billion parameters,} \end{array}$$

which is the same order of magnitude at which ESM-2 models start to taper off in their improvement (roughly 3 billion parameters) (17). A model that segments a protein into common motifs, as our work suggests pLMs are doing, offers a clear route to compression. A downside of such compression is that within-family evolutionary effects such as multiple stable conformations are inaccurately predicted by ESM-2 (*SI Appendix*), a clear area for future improvement.

In summary, our work has demonstrated how a fundamentally powerful unsupervised learning approach—that of masked language modeling—enables storing coevolutionary statistics agnostically for thousands of protein families. Although they have not yet reached the ability to directly model the physics of protein folding, we anticipate that this research and other ongoing interpretability studies will shed light on how we might actually use deep learning to approximate the fundamentals of biophysics.

## Materials and Methods

### Isoform Dataset Curation and Analysis.

We collected examples of isoforms identified previously (11–14) as cases where splicing would disrupt ordered domains, along with associated structures. For each, we identified a corresponding isoform and full-length protein in UniProt. We predicted structure models in AlphaFold2 (1) using ColabFold (30); OmegaFold (2) using the OmegaFold notebook available at https://colab.research.google.com/github/sokrypton/ColabFold/blob/main/beta/omegafold.ipynb; and ESMFold (4) using the ESMFold server available at https://esmatlas.com/resources?action=fold.

We calculated the SAP score of isoform models using the “per_res_sap.xml” script from *SI Appendix* of ref. 31. We calculated RMSD between structure models of isoforms and the full-length experimental structure in PyMOL (32) for α-carbons for aligned regions. Aligned regions were manually determined from alignments of each isoform to each full-length sequence using the “global alignment with free end gaps” setting and the BLOSUM62 matrix in the Geneious Prime software.

### Dataset for Model Comparison and Contact Recovery.

We obtained 2,245 structures from the Generative Regularized ModeLs of proteINs (GREMLIN) Coevolution predictions database for PDB_EXP with more than 1,000 sequences in MSA (19). Similar structures were filtered based on a TMalign (33) score exceeding 0.5. We used proteins with a length from 200 to 600 amino acids to ensure a similar size across proteins and enough length for exploring the effect of flanking region (*SI Appendix*, Fig. S6*A*). We selected only proteins that were missing fewer than 50 residues in the structure to ensure the majority of residues are present in the experimental structure. With these filtering steps, we obtained a dataset of 1,431 proteins in total.

### Weights and Contact Maps from a Linear Model.

To calculate pairwise coupling weights and contact maps from MSAs via a linear model, we use the inverse covariance method presented in ref. 25. Dauparas et al. demonstrated that MRF and MG models can be mapped to the same graphical model representation (25). The main difference is that MRFs consider the tokens in biological sequences as categorical, and MGs approximate them as continuous variables. Estimating a set of pairwise coupling weights $W^{L,A,L,A}$ for either thus depends primarily on the loss function used. Dauparas et al. demonstrated empirically that the following estimation, which derives from a mean-squared-error loss for an MG formalism, performs comparably to cross-entropy loss in an MRF, as used in GREMLIN ref. 18 and other models, but with substantially less compute. We defer the reader to ref. 25 for the complete derivation.

The MSA sequences are filtered with HHfilter (34) based on a sequence identity cutoff of 90% to reduce sequence redundancy and coverage of 75% to remove those with too many gaps (35). The sequences are one-hot-encoded and written in the form $X\in R^{N\times LA}$, where N is the number of sequences, L is the sequence length, and A is the number of letters available in the alphabet (A=20 for proteins). The pairwise coupling weights W are calculated as

[4] $$\begin{array}{c} W=-{\left(\;\text{Cov}\left(\hat{X}\right)+\frac{4.5}{\sqrt{N}}I\right)}^{-1}, \end{array}$$

where $\hat{X}$ is the mean-centered MSA, i.e. $\hat{X}=X-\bar{X}$, and $\bar{X_{n,jk}}=\frac{1}{N}\sum_{i=1}^{N}\bar{X_{i,jk}}$ for all n. The $\frac{4.5}{\sqrt{N}}$ term is introduced for shrinkage and is empirically estimated in ref. 25. Note that the expression for W above is technically the weight matrix minus the identity matrix. This is expressed in ref. 25 as W=W+I, but this makes no difference in calculation.

We calculate a contact matrix $C\in R^{\mathrm{LxL}}$ with entries $c_{\mathrm{ij}}$ from the above tensor W using

[5] $$\begin{array}{c} c_{\mathrm{ij}}=\;\text{APC}\left({\left(\sum_{n=1}^{20}\sum_{m=1}^{20}W{\left[i,n,j,m\right]}^{2}\right)}^{1/2}\right), \end{array}$$

where APC is the average product correction (36). For a matrix in $R^{L\times L}$ composed of entries $m_{\mathrm{ij}}$, we calculate the APC as

[6] $$\begin{array}{c} \;\text{APC}\left(i,j\right)=m_{\mathrm{ij}}-\frac{\sum_{i^{\prime }=1}^{L}m_{i^{\prime }j}\sum_{j^{\prime }=1}^{L}m_{ij^{\prime }}}{\sum_{i^{\prime }=1}^{L}\sum_{j^{\prime }=1}^{L}m_{i^{\prime }j^{\prime }}}. \end{array}$$

### Calculation of Long-Range Contact Prediction Accuracy.

We evaluated the contact prediction performance based on the precision of the top L/2 (L is the length of the protein) predicted long-range contacts (separated by more than 24 residues) by confidence (4). The contacts from experimental structures were identified based on the criterion that two amino acids have Cα-pagination distance < 10 Å.

### Language Model Contact Map via Jacobian.

To calculate the categorical Jacobian of a language model, each of L positions in a sequence is mutated to all A possible tokens (for proteins, A=20) and input into the language model to predict the resulting logits across the entire sequence, where the logits are shaped L×A. The difference between the logits of the original sequence and the logits of the mutated sequences was calculated to get the Jacobian matrix.

Written formally, we define the categorical Jacobian J for a protein language model f(X), which accepts as its input a protein sequence X with length L and alphabet size A, as

[7] $$\begin{array}{c} J=\left[\begin{array}{ccc} f\left[X\left(x_{1}\to a_{1}\right)\right]-f\left[X\right] & \cdots & f\left[X\left(x_{1}\to a_{20}\right)\right]-f\left[X\right]\\ \vdots & \ddots & \vdots \\ f\left[X\left(x_{L}\to a_{1}\right)\right]-f\left[X\right] & \cdots & f\left[X\left(x_{L}\to a_{20}\right)\right]-f\left[X\right] \end{array}\right]. \end{array}$$

Above, f[X] is the original logits output by the language model, a matrix of logits with size L×A. $f[X\left(x_{i}\to a_{n}\right)]$ represents the logits returned when position i has been mutated to token $a_{n}$. The Jacobian is therefore a tensor with size [L,A,L,A]. J is mean-centered and symmetrized. We obtain a contact map from J analogously to Eqs. **5** and **6**. It can be shown that applying the same categorical Jacobian operation to an MRF or MG model will return the pairwise weights matrix W.

To evaluate the correlation between the pairwise couplings from ESM-2 models and the couplings from a linear model, we first selected the top-weighted L interresidue contacts from the contact map calculated from the linear model (following average product correction). This results in L × L × 20 × 20 weights from both the linear model couplings (W) and the ESM-2 jacobian J. We expect many of these weights to be close to zero and not meaningful, so we calculate Spearman correlation over a range of cutoffs filtering values close to zero.

If $\hat{W}$ is this reduced mean-centered set of couplings from the linear model, and $\hat{J}$ is the reduced mean-centered ESM-2 jacobian, we calculate the Spearman correlation over the subset of $\hat{W}$ and $\hat{J}$ whose absolute value is greater than b SDs of $\hat{W}$, where b∈[0,4]. The Spearman correlation is calculated in SciPy (37).

### Recovery of Contact with Increasing Flanking Region.

For contact recovery between segments closer in distance, we created the following procedure to account for the fact that ESM-2 was masked to not predict contacts between residues closer than 6 residues apart. We scanned over the contact map output by the ESM2 Contact Head for pairs of segments (each 11 aa in length) whose ends are separated by 5 aa (i.e., the centers of the segments are separated by 15 aa). We only selected segment pairs that have extensive contacts based on:

[8] $$\begin{array}{c} \;\sum_{i=m}^{m+10}\sum_{j=n}^{n+10}a_{\mathrm{ij}}>10, \end{array}$$

where $a_{\mathrm{ij}}$ is the contact probabilities corresponding to positions i,j from the LM contact of the sequence.

For each of the 1,431 proteins, we sampled at most 3 segments. We randomly picked the first segment pair and then selected the next two pairs by choosing the pairs that were the furthest from the already selected pairs. In total, 4,022 segment pairs from 1,429 proteins were examined.

For examining the interaction between pairs of segments that are further apart, we chose pairs of SSEs with centers separated by at least 50 amino acids, because the contact probability derived from the GREMLIN dataset showed correlations diminished beyond 40 residues (*SI Appendix*, Fig. S6*B*). To extract secondary structures, we predicted structures using ESMFold (4) and then used Python dictionary of Secondary Structure of Protein (PyDSSP) (38) to calculate secondary structure. To standardize the lengths of SSE segments, we took the centers of SSEs and selected 5 residues on both sides of the center. We selected SSE segment pairs with more than 10 residues to the protein termini. We again selected only SSE pairs with extensive contacts. We randomly sampled maximally 3 segments per protein. For SSE segment pairs separated by 50 to 100 residues, 1,273 segment pairs from 821 proteins were examined. For SSE segment pairs separated by more than 100 residues, 304 pairs from 266 proteins were examined.

After selecting the segment pairs, we compared the recovery from unmasking the region flanking the outer sides of the segment pairs and from random unmasking. We conducted the unmasking in three different ways: 1) symmetrically increase the unmasked residues flanking each of the outer sides of the segment pairs, 2) randomly unmask an increasing number of residues, 3) randomly unmask residues but avoid the nearest 30 aa around the ends of the segment pairs.

The contact recovery was calculated via

[9] $$\begin{array}{c} \;\text{Recovery}=\sum_{i,j}\frac{a_{\mathrm{ij}}b_{\mathrm{ij}}}{a_{\mathrm{ij}}^{2}}, \end{array}$$

where $a_{\mathrm{ij}}$ and $b_{\mathrm{ij}}$ are the contact probabilities corresponding to positions i,j from the LM contact of the original and masked sequences, respectively. When the score was higher than 0.5, we regarded it as a recovery of contact.

During ESM-2 training, Beginning-of-sequence (BOS) and End-of-sequence (EOS) tokens are used to indicate the start and end of the protein for the model to distinguish a full-sized protein from a cropped one. For recovery experiments in Fig. 6 and *SI Appendix*, Figs. S2–S4, we replaced these tokens at the start and end of each protein with a mask token.

### Analysis of Step-Function Type Behavior of Contact Recovery Experiments.

Different behaviors in contact recovery were characterized in the following way. For segments that ultimately achieved contact recovery higher than 0.5, we calculated the maximal recovery increase over a flank length increase of 1 residue. A cutoff of 0.5 was used to define an “abrupt” change in recovery. To examine the cases where a sudden increase of recovery was achieved when a certain residue was included, we analyzed recovery increase v.s. the number of unmasked flanking residues, and we noticed that a small number of segment pairs have a lot of fluctuations. We filtered out these cases based on the criteria that fewer than 3 residues in the next 10 residues after the “jump in recovery” have a drop in recovery value of 0.2. For the cases where there was a “jump in recovery” and the recovery values stayed relatively stable after the “jump,” we evaluated the total number of unmasked residues needed for each segment pair. We also examined the minimum number of unmasked residues needed by asymmetrically unmasking the outer flanking region. The examples of different recovery curves and the detailed filtering process are shown in *SI Appendix*, Figs. S2–S4.

## Supplementary Material

Appendix 01 (PDF)

## Data Availability

The dataset of 18 isoforms and scripts to perform analysis are available at https://github.com/HWaymentSteele/Isoforms_benchmark_2024 (15). The code for categorical Jacobian and contact prediction analyses is available at https://github.com/zzhangzzhang/pLMs-interpretability (23). The modified positional embedding version of ESM-2 and ESMFold are available at https://github.com/garykbrixi/esm_gap_distance (39). Interactive Google Colab notebook for extracting conservation and coevolution (categorical Jacobian) from ESM models is available at https://colab.research.google.com/github/sokrypton/ColabBio/blob/main/categorical_jacobian/esm2.ipynb (24).

## Footnotes

^*^Storing sequence conservation requires (20,000 protein families × 256 sequence positions × 20 amino acids) = 100 million parameters for any model.
