# Supplementary material for: Protein language models learn evolutionary statistics of interacting sequence motifs
Source: Proc Natl Acad Sci U S A. 2024 Oct 28;121(45):e2406285121. doi: 10.1073/pnas.2406285121 (PMC11551344; doi:10.1073/pnas.2406285121)
Supplement: Supplementary file 1 — Appendix 01 (PDF) [file pnas.2406285121.sapp.pdf]

PNAS

Supporting Information for

Protein language models learn evolutionary statistics of interacting sequence motifs

Zhidian Zhang, Hannah K. Wayment-Steele, Garyk Brixi, Haobo Wang, Dorothee Kern, Sergey Ovchinnikov

Sergey Ovchinnikov
E-mail: so3@mit.edu

This PDF file includes:

Supporting text
Figs. S1 to S17
Table S1
SI References

Supporting Information Text

Analyzing categorical Jacobian of various ESM models. For a more extensive analysis with different step sizes, eigenmodes removal, and models, we used a dataset of 383 proteins from distant families (1). For all models, increasing the step size to h = 1.0 improved contact prediction accuracy (Fig. S7). Removing 1 eigenmode resulted in the optimal contact accuracy for inverse covariance and ESM models (Fig. S8). In terms of contact prediction accuracy of different models, ESM3 has higher accuracy than ESM1 and ESM2, and ESM2 of increasing size has increasing accuracy (Fig. S9).

Calculating contacts by mutating each residue to the `mask` token instead of 20 aa types resulted in a decrease in accuracy. We also analysed the contact prediction accuracy of calculating the categorical Jacobian from mutating each residue to the `mask` token. The mean P@L decreased from 0.80 to 0.76 (Fig. S10).

Masking the `BOS` and `EOS` tokens reduced the context needed for contact recovery. Replacing `BOS` and `EOS` tokens at the start and end of each protein with a `mask` token reduced the amount of context required for contact recovery (Fig. S11).

Masking and removal + offset had similar performance in contact recovery. Instead of masking, positions can also be removed and the residue index can be offset to account for the removal (Fig. S12A). We find removal+offset to result in a decrease in segment pair recovery performance (Fig. S12B). This might be caused by the inpainting of residue contacts for residues substituted by `mask` token.

Method: We modified the rotary position embeddings (RoPE) (2) to allow modifying the positions of the amino acids without adding additional tokens between them. We achieved this by extending the length of the sine and cosine tables and retrieving the desired index for each amino acid from the sine and cosine tables. This way, the total length of the transformer input and operations stayed the same, but the RoPE index of each query and key corresponded to the offsets.

Strand-strand interactions demanded less context compared to helix-helix interactions. We found that strand-strand interactions demanded less context compared to helix-helix interactions (Fig. S13). This is consistent with the notion that β-strands are frequently composed of fewer residues than α-helices, and that β-strands tend to form stable β-sheets. This difference in the context needed for recovery for helices and strands was much more pronounced when using offset compared to masking (Fig. S13).

ESM2 predicted same fold for two KaiB sequences with different experimental structures. KaiB is a metamorphic protein that adopts two distinct states as part of its function. We extracted contacts for constructs demonstrated in vitro to be stabilized for both states with ESM2. While experimental results showed that each sequence favors one state over another, ESM2 predicted that both sequences fold to the thermodynamically unfavorable fold-switched (FS) state (Fig. S14). ESM2 showed the same failure as the previous MSA-based methods such as MSA transformer and AF2 with default settings (3).

ESM2 predicted the same interchain contacts for bacterial response regulator subfamilies with diverse interchain contacts. Three bacterial response regulator subfamilies were shown to have diverse interchain contacts between their homomeric interfaces despite having similar intrachain contacts (4). This provides an incisive example of a potential pitfall of evolutionary models, where the properties of the protein family lead to a prediction which does not match the property of the sequence. If an evolutionary model includes proteins from the different subfamilies together, it will erroneously predict contacts which do not exist for that sequence.

We observed that the ESM2 predictions for 1NXS and 4CBV included contacts found only in the other families while predictions and only a small fraction of correct family-specific contacts were recovered. 4CBV predictions did not include any correct family-specific contacts while 1NXS had 7 4E7P specific contacts predicted, more than the 3 which were predicted when using the actual 4E7P sequence (Fig. S15).

This type of failure is similar to the outputs of MSA-based methods, which cannot differentiate different subfamilies if the aligned sequences mix together different subfamilies with different properties.

Method: We calculated the interchain contacts from experimental structures of the three different families (contact defined as < 12 Å between alpha carbons) and predicted contact maps for them using the sequence of the domain and the ESM2 contact head. The outputs of the ESM2 contacts on the different aligned using US-align, removing all gap positions. We consider the top 300 contacts predicted by ESM2 which are >5 positions off the diagonal. Interchain contacts which are unique to each structure are colored according to the family they belong to in the distogram.

Comparison of categorical Jacobians of sequences from the same MSA. We hypothesized the categorical Jacobian to be identical for all sequences of the protein families, unless sub-clusters of the family have differing constraints. To test for this, we picked 10 random protein families shorter than 300 residues and with a wide range of sequence identities to the query sequence and compared the categorical Jacobians from ESM2-3B.

We binned sequences based on different sequence identities, the width of bins is 0.1, and we randomly selected 3 sequences from each protein. We compared the categorical Jacobian matrices and contact matrices of proteins corresponding to the aligned part of the sequences and calculated the mean Spearman correlation. For Spearman correlation calculation of categorical Jacobian matrices, we followed the same procedure described in the method section of the main text.

We observed that for ESM2 the similarity in contact (Fig. S16A) and categorical Jacobian prediction (Fig. S16B) is correlated with the sequence identity of sequences from the same MSA.

Comparison with the contact extraction approach from Trinquier et al. (5). Trinquier et al (5) used all combinations of pairs of mutations in addition to single mutations to compute contacts. Due to the extensive computation required, we are not able to do a comprehensive comparison between the approaches. Here we show one example using the ESM2-150M model, which took 3 days to compute on a single A6000 GPU (Fig. S17). We compared the pairwise dependencies and contact maps extracted using the two methods for 50S ribosomal protein L29 from E. coli. Though the results are not identical, the categorical Jacobian is highly correlated to this much more expensive calculation.

	Pair separation: 15	50-100	>100
**Flank**	32	44	60
**Random**	100	104	156
**Random, avoid nearest 30 aa**	110	122	166
**Ratio, "Random" to "Flank" context**	3.1	2.4	2.6

Table S1. Context needed for 50% of segment pairs to have a contact recovery score of 0.5.

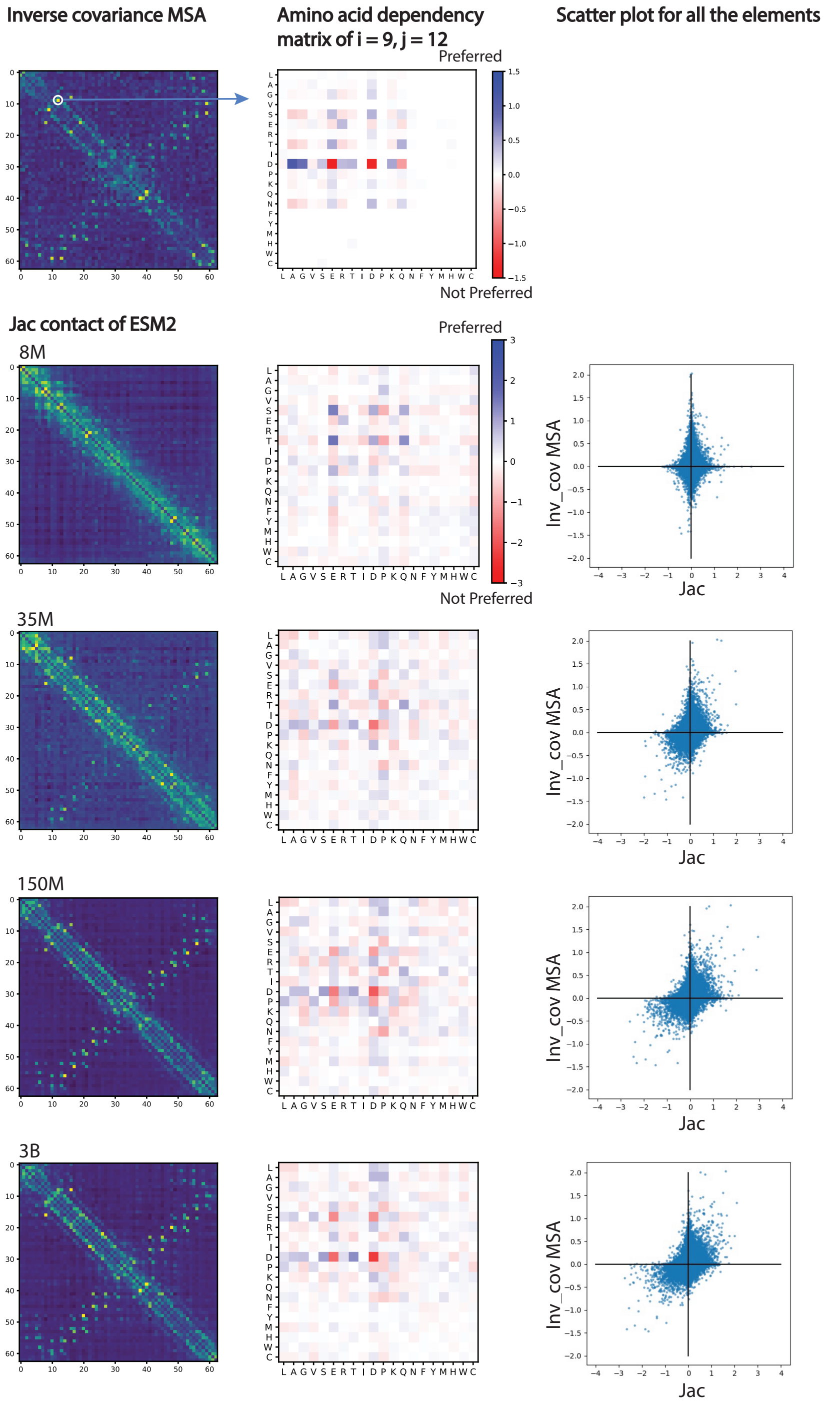

Fig. S1. The Jacobian contact maps, amino acid dependency plot at i = 9 and j = 12, and the scatter plot for all elements of ESM models of different sizes showed that larger ESM2 models captured evolution information better.

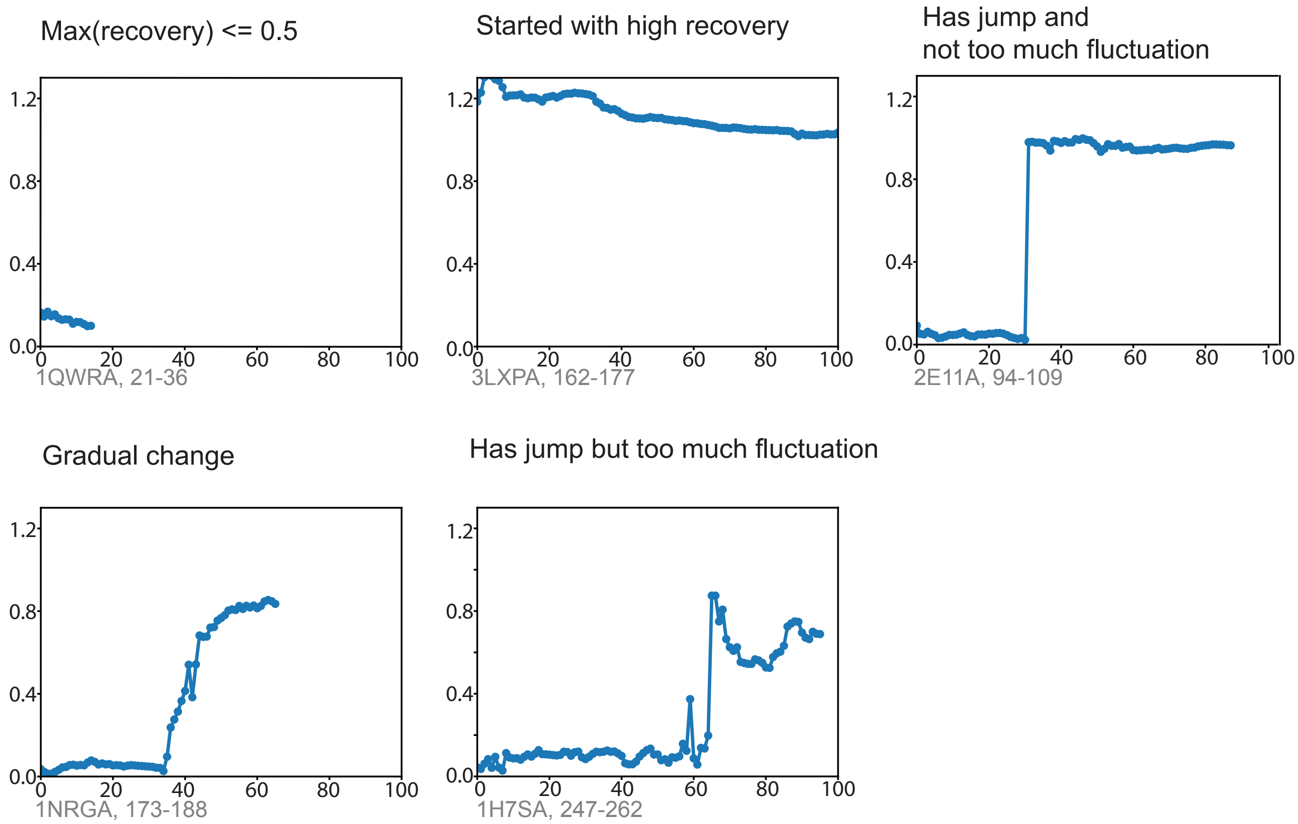

Fig. S2. Different types of segment contact recovery curves.

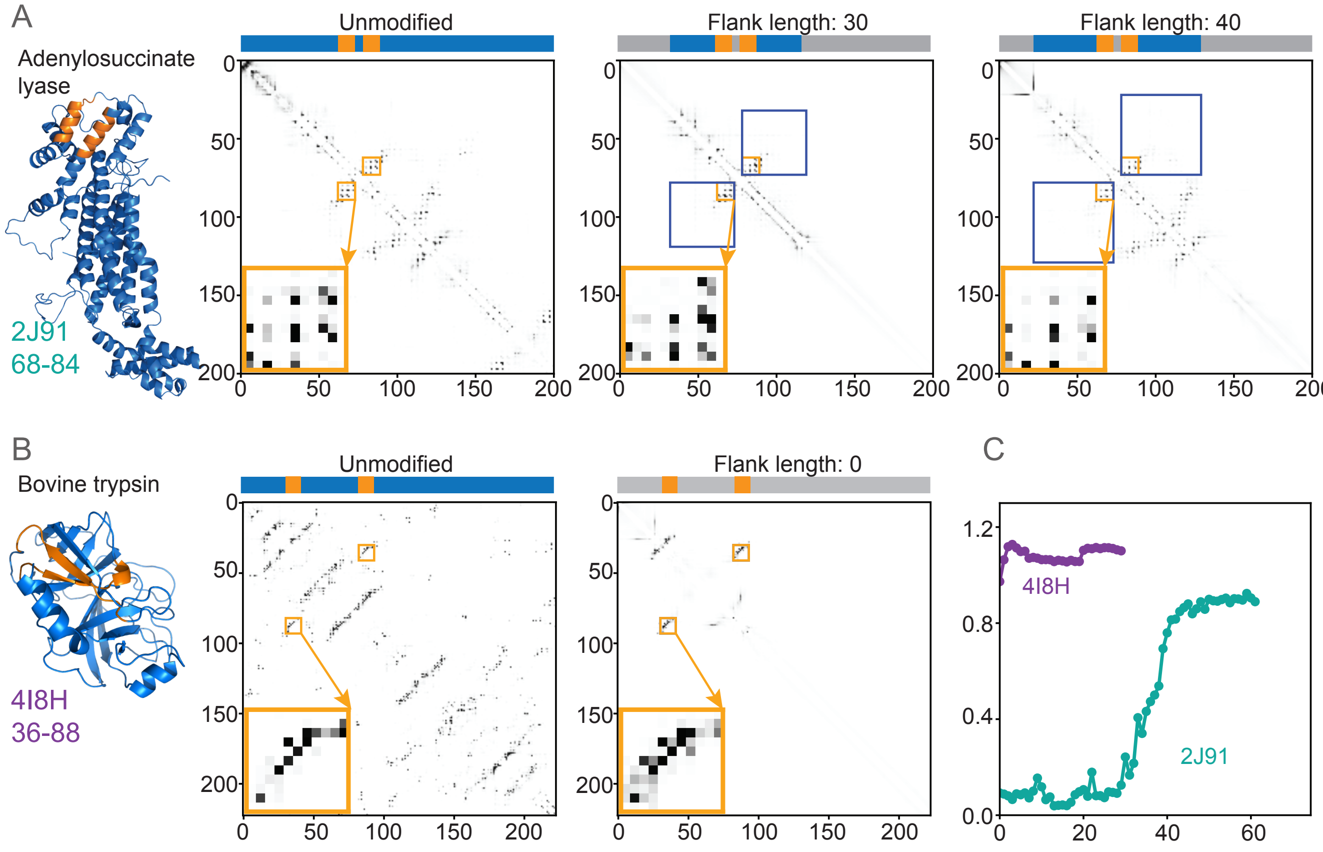

Fig. S3. ESM2 contact maps of fully unmasked and partially unmasked sequences from (A) adenylosuccinate lyase (PDB 2J91) (6) and (B) bovine trypsin (PDB 4I8H) (7). (C) A gradual increase was observed in the recovery of contacts between two nearby α-helices segments in the adenylosuccinate lyase (PDB 2J91) protein with an increasing amount of unmasked flanking residues. In the bovine trypsin (PDB 4I8H), the contacts between two β-strands with centers separated by 52 residues were recovered without unmasking any flanking residues.

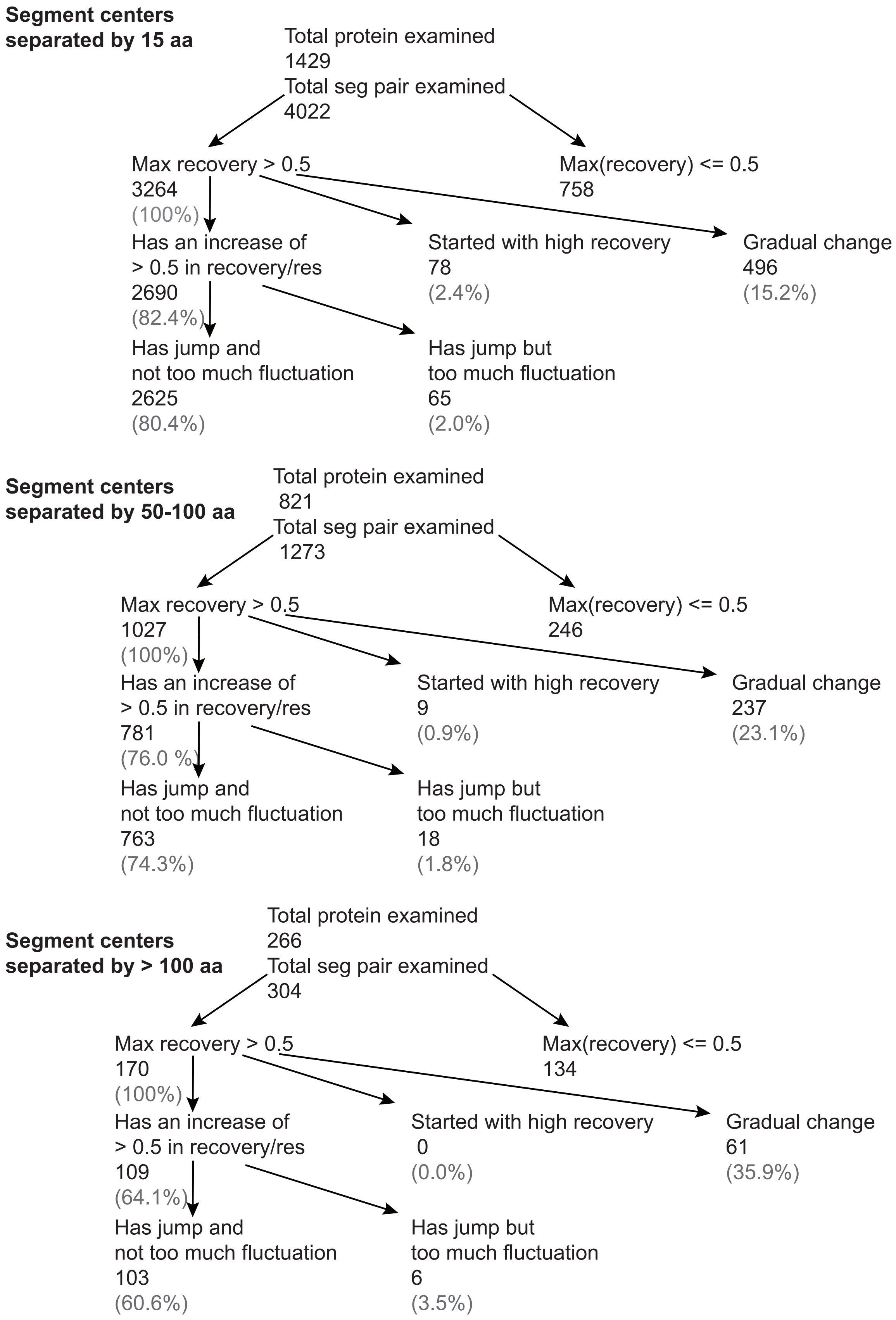

Fig. S4. The classification process and prevalence of different types of segment contact recovery curves.

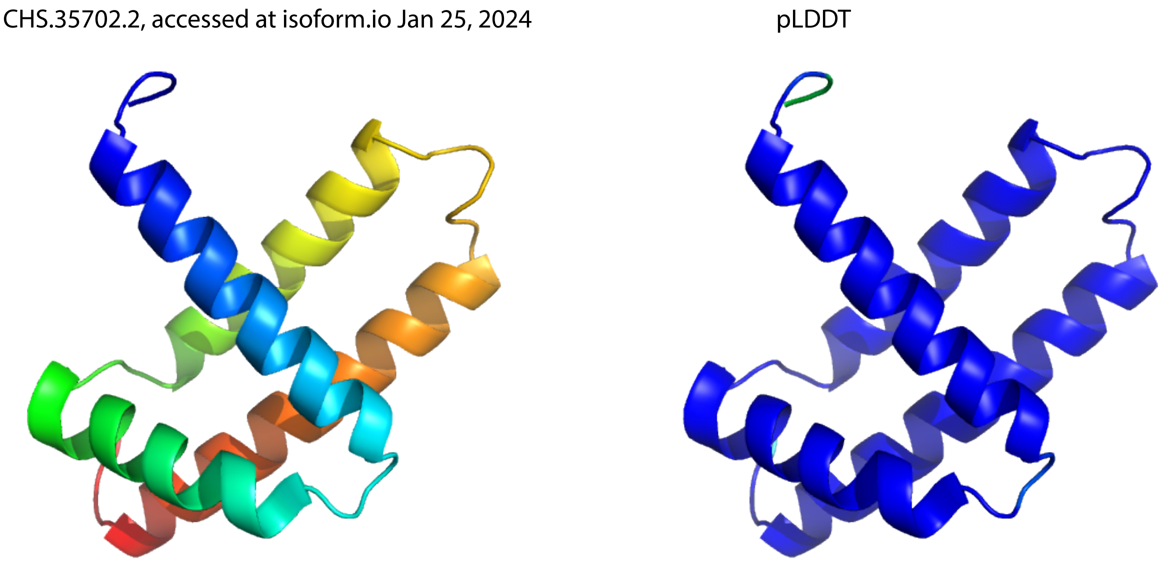

Fig. S5. Erroneous prediction for myoglobin isoform CHS.35702.2 is present in isoform.io database, accessed January 2024. (cf. Fig. 2A).

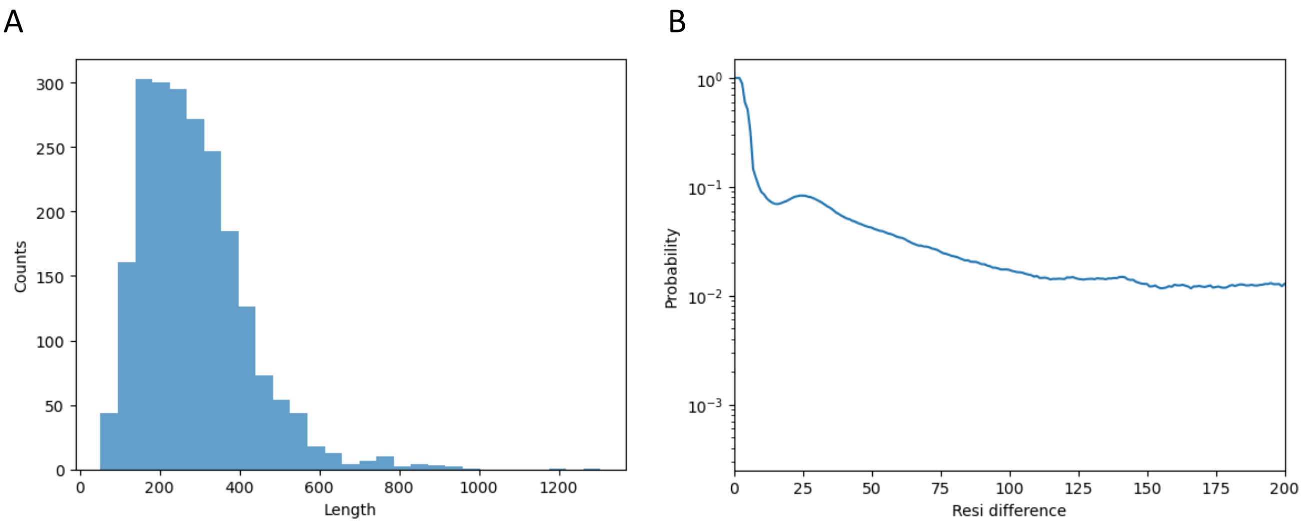

Fig. S6. (A) Size distribution of proteins in the dataset. (B) Probability of contact between residues separated by different distances.

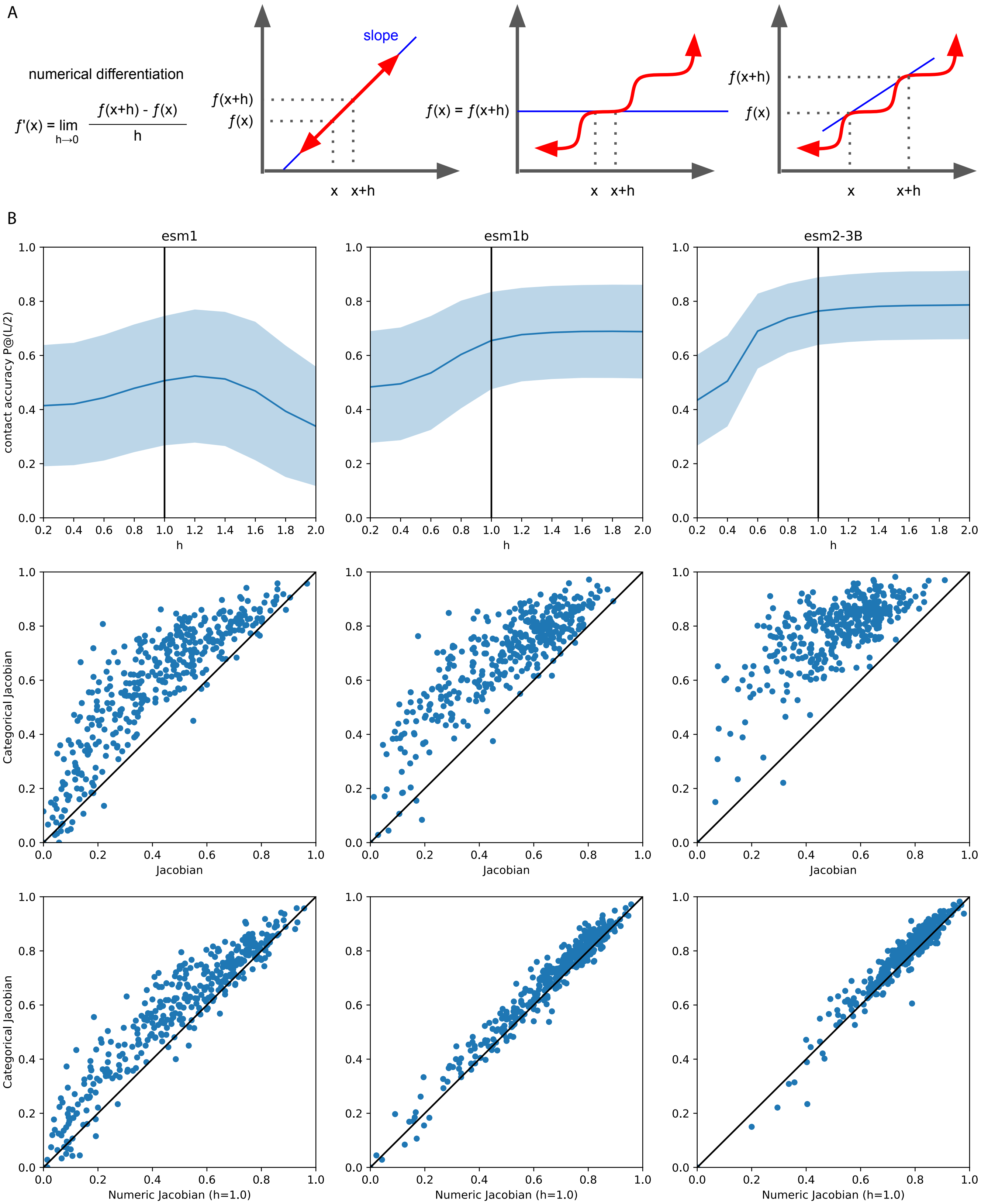

Fig. S7. (A) Scheme demonstrating why in a linear model, perturbation of any step size returns the same value in the Jacobian, while in ESM-2, a small perturbation to the one-hot encoded input is insufficient to perturb the output, yet increasing the step size improves contact map accuracy. (B) Contact prediction accuracy of Jacobian with different step sizes (h). (C) Comparison of contact prediction accuracy of categorical Jacobian and Jacobian. (D) Comparison of contact prediction accuracy of categorical Jacobian and Jacobian with a step size of 1.0.

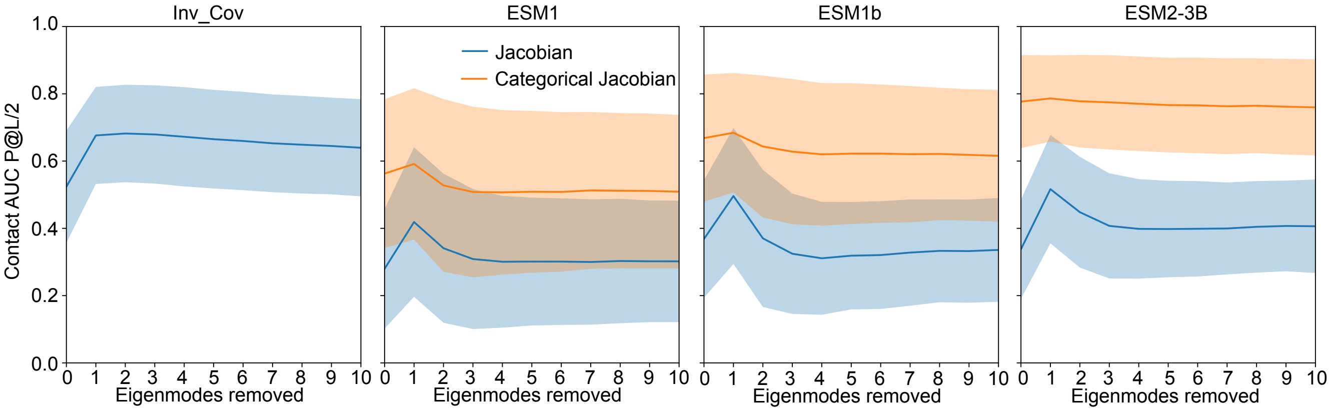

Fig. S8. Contact prediction accuracy of inverse covariance and ESM models with different eigenmodes removal.

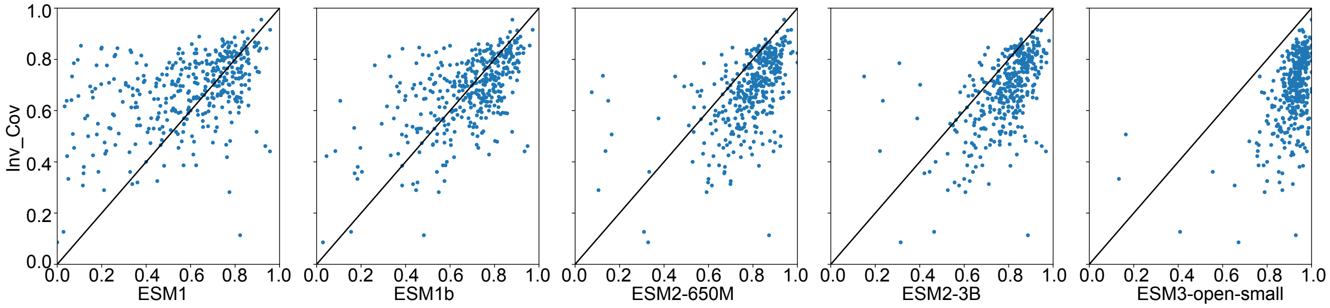

Fig. S9. Comparison of contact prediction accuracy from inverse covariance and categorical Jacobian of different ESM models.

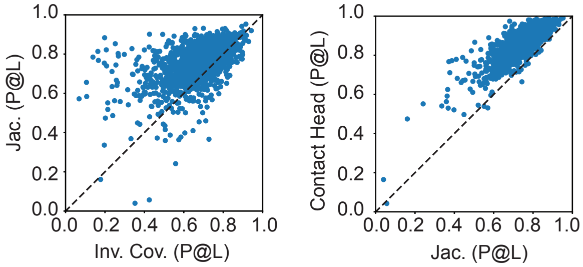

Fig. S10. Contact prediction accuracy of categorical Jacobian by mutating each residue in sequence to the `mask` token.

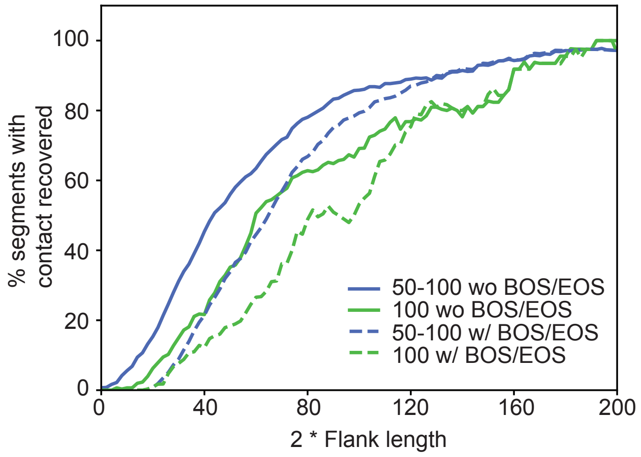

Fig. S11. The recovery of contacts from partially masked sequences required less unmasked flanking regions without `BOS` and `EOS` compared to with `BOS` and `EOS`. N = 1273 and 304 for segments separated by 50-100 aa and > 100 aa

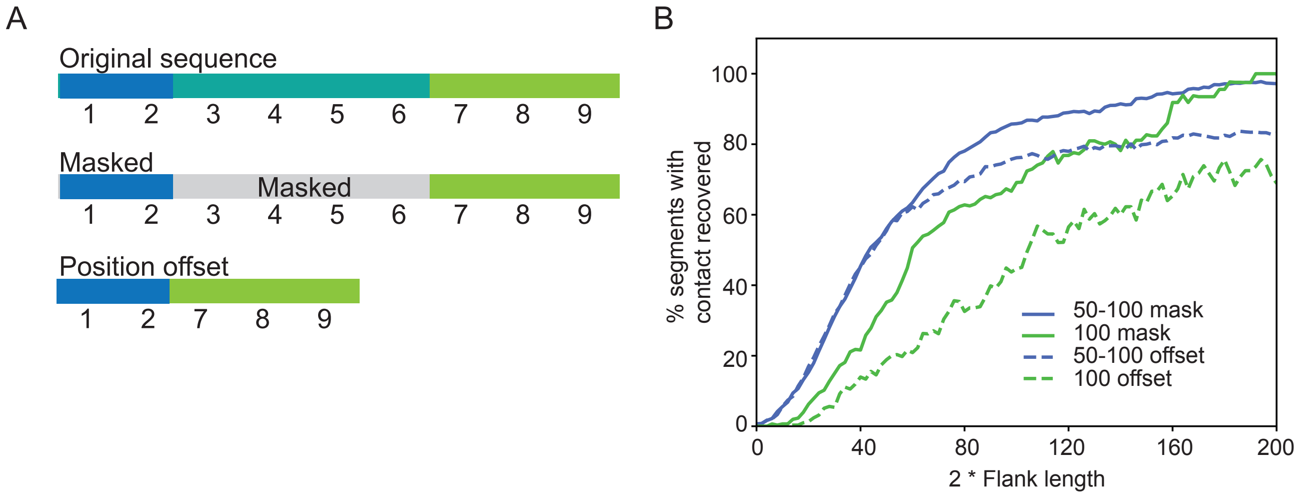

Fig. S12. (A) Scheme depicting masking and position offset. (B) Masking resulted in higher recovery of SSE pair contacts compared to offset. All experiments were done without BOS and EOS. N = 1273 and 304 for segments separated by 50-100 aa and > 100 aa.

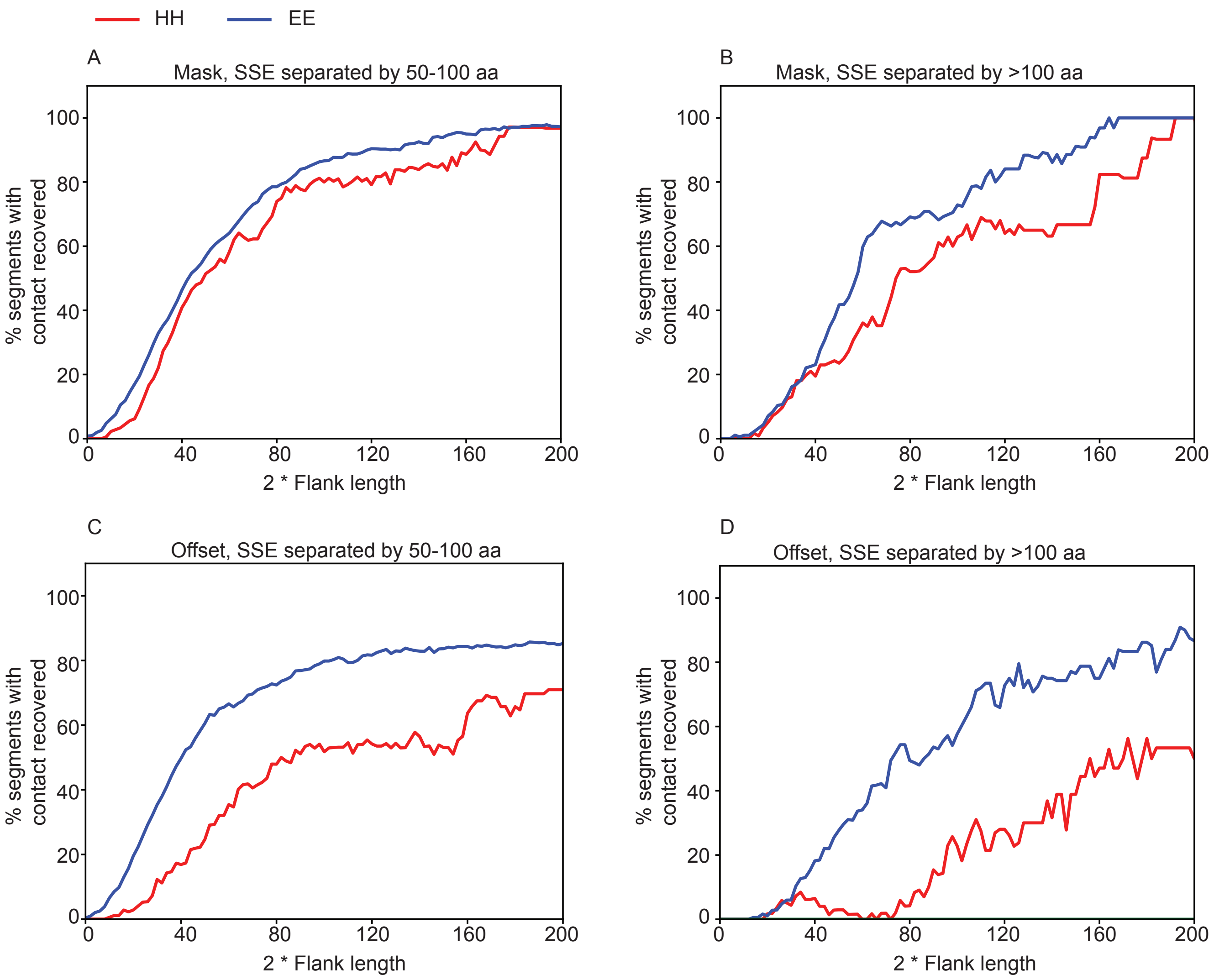

Fig. S13. Recovery of strand-strand contacts required less flanking region than helix-helix contacts for contact recovery experiments done with (A) masking and SSE pairs separated by 50-100 residues, (N = 178 for HH, N = 1082 for EE) (B) masking and SSE pairs separated by >100 residues, (N = 118 for HH, N = 184 for EE) (C) position offset and SSE pairs separated by 50-100 residues, (N = 178 for HH, N = 1082 for EE) (D) position offset and SSE pairs separated by >100 residues (N = 118 for HH, N = 184 for EE). All experiments were done without `BOS` and `EOS`.

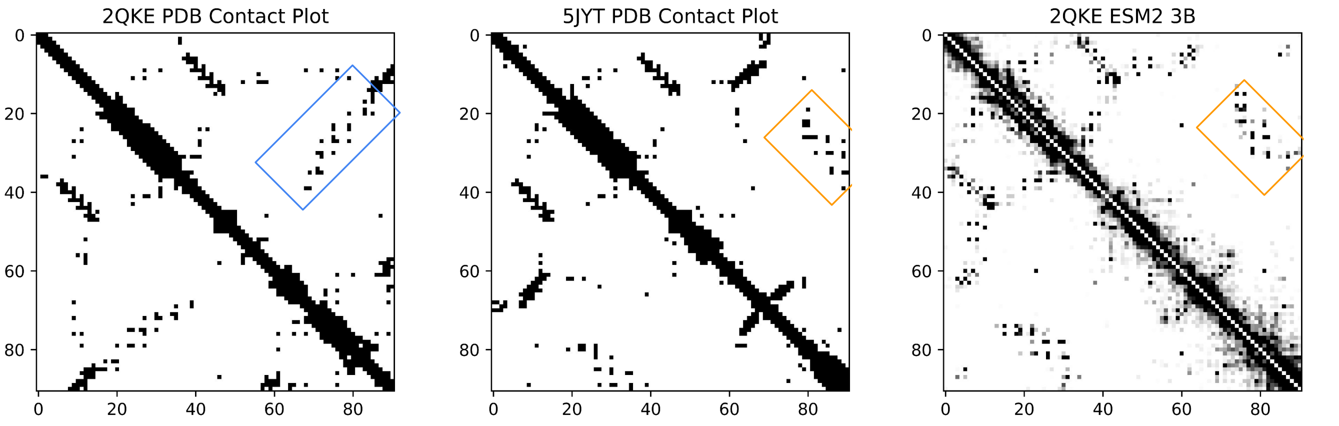

Fig. S14. KaiB from *Thermosynechococcus elongatus* thermodynamically favors the Ground state represented in (PDB: 2QKE, distinguishing features boxed in blue), yet also samples the thermodynamically unfavored fold-switched (FS) state (PDB: 5JYT, distinguishing helix-helix interaction boxed in orange). Contact predictions from ESM2 correspond predominantly to the FS state.

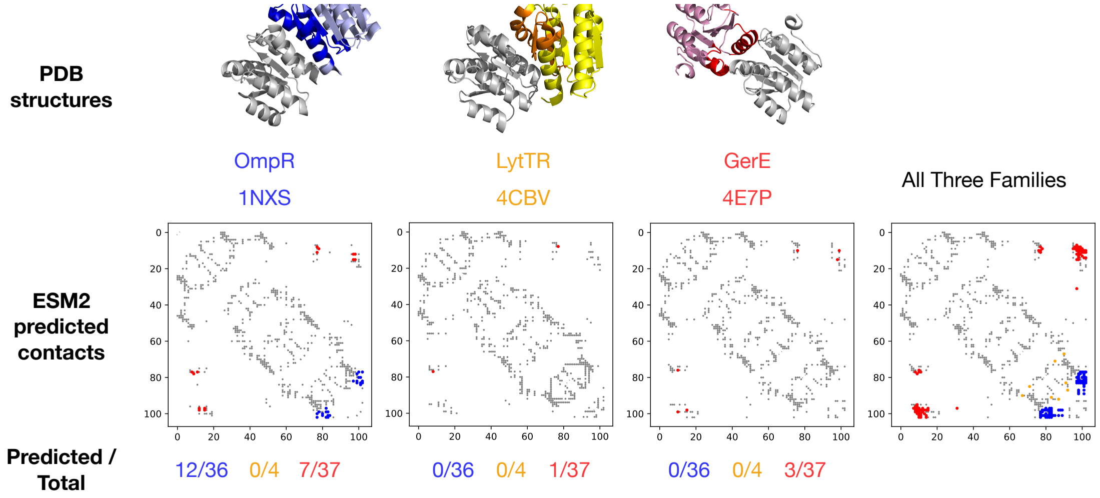

Fig. S15. ESM2 predicted contacts from the three different full protein sequences compared to the unique family-specific interchain contacts. PDB structures show the correct interchain contacts for each family. Unique intercontacts which are predicted by ESM are colored according to their family on the distogram. ESM2 erroneously predicts family-specific interchain contacts from the other families and misses many family-specific contacts. The number of interchain residue contacts predicted over the total number of family-specific contacts is displayed below.

A

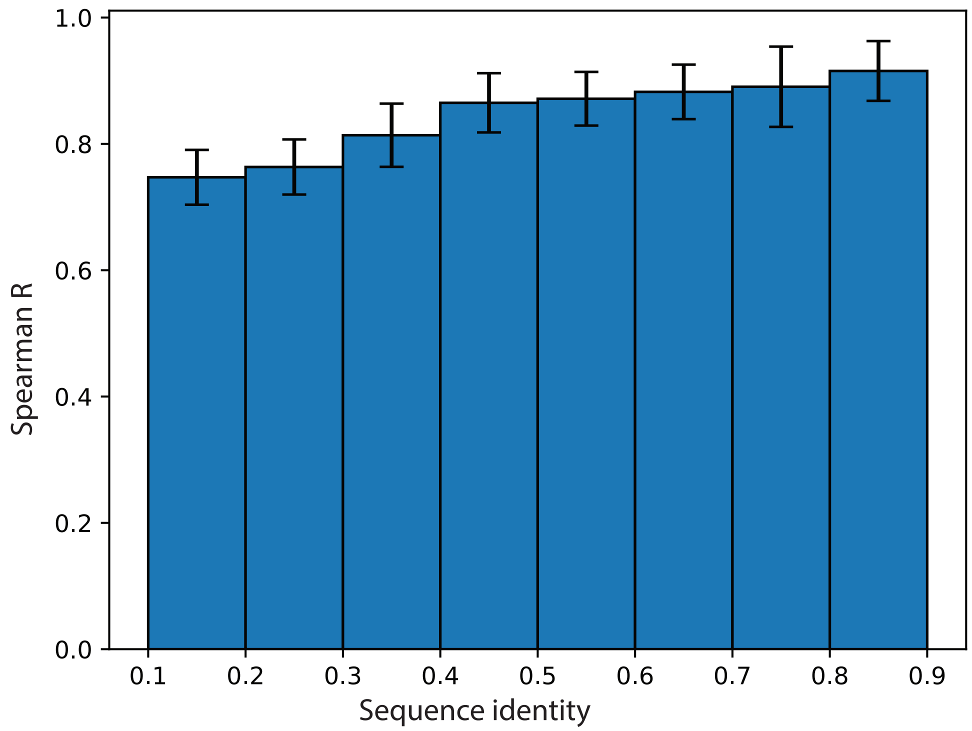

B

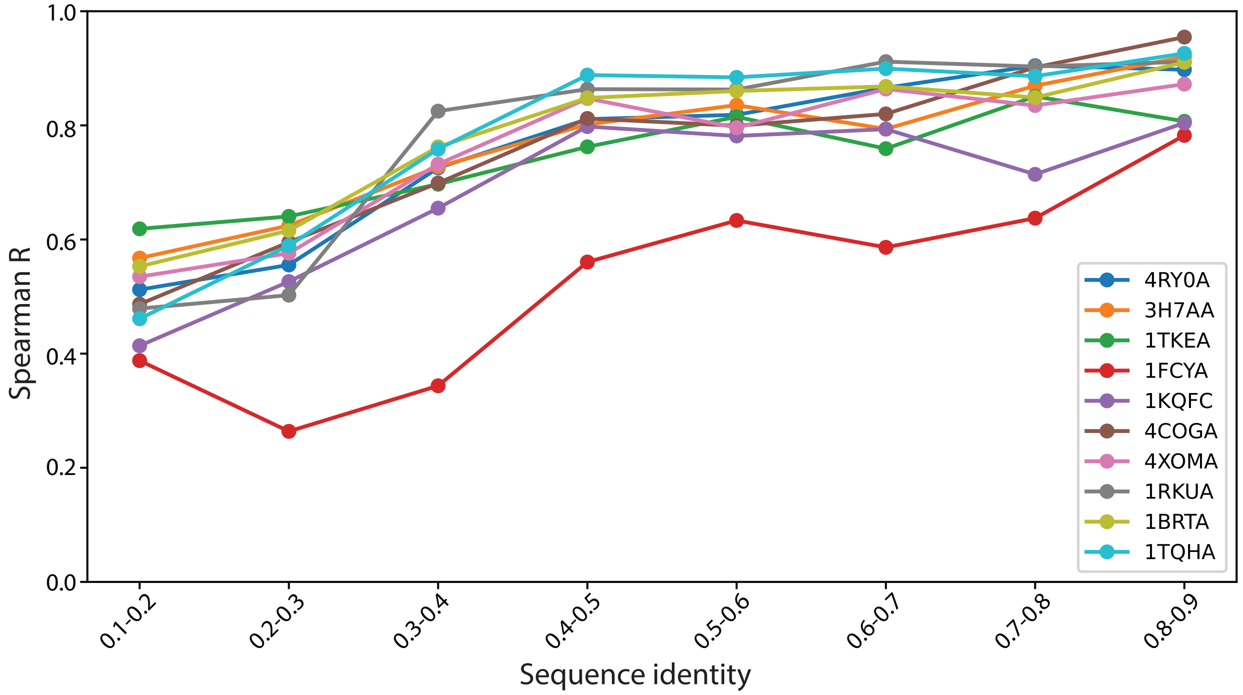

Fig. S16. Spearman correlation for (A) the contact map calculated from the categorical Jacobian and (B) the categorical Jacobian of sequences from the same MSA.

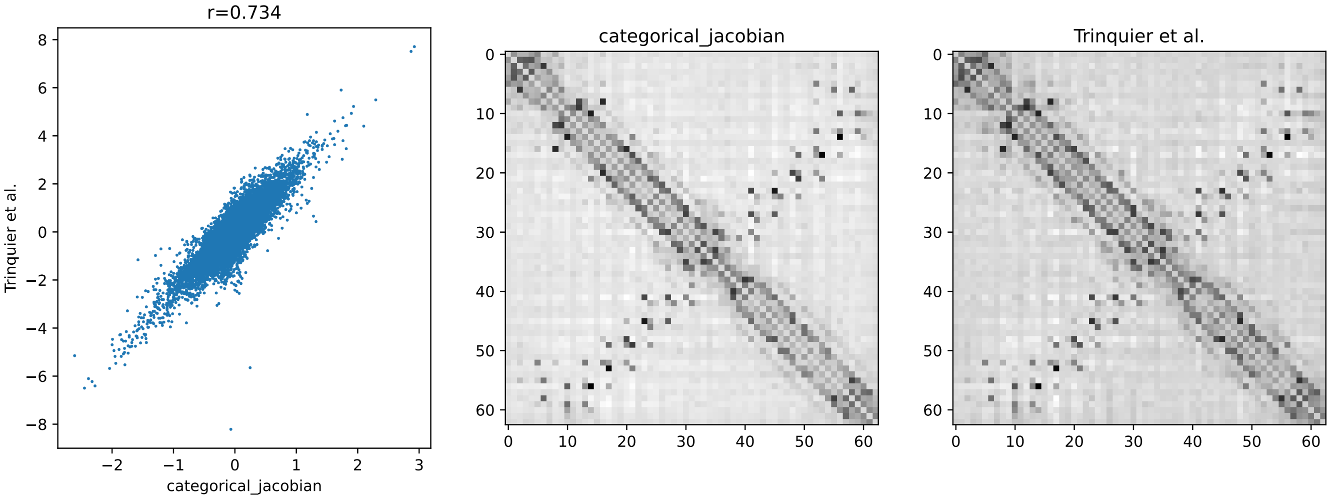

Fig. S17. Comparing pairwise dependencies extracted using method proposed by Trinquier et al. to categorical jacobian. Analysis is shown for 50S ribosomal protein L29 from E. coli. The first plot compares all the values in LxLxAxA tensor between the methods, after centering, symmetrizing and zeroing diagonal. The next two plots shows the contact map after L2 normalization over AxA dimensions, and average product correction.

References

1. J Dauparas, et al., Unified framework for modeling multivariate distributions in biological sequences (2019).
2. J Su, et al., RoFormer: Enhanced Transformer with Rotary Position Embedding. (2021).
3. HK Wayment-Steele, et al., Predicting multiple conformations via sequence clustering and AlphaFold2. *Nature* **625**, 832–839 (2024).
4. Malinverni, Barducci, Coevolutionary Analysis of Protein Subfamilies by Sequence Reweighting. *Entropy* **21**, 1127 (2019).
5. J Trinquier, G Uguzzoni, A Pagnani, F Zamponi, M Weigt, Efficient generative modeling of protein sequences using simple autoregressive models. *Nat. Commun.* **12**, 5800 (2021).
6. P Stenmark, Moche, M., Arrowsmith, C., Berglund, H., Busam, R., Crystal Structure of Human Adenylosuccinate Lyase (2006).
7. D Liebschner, M Dauter, A Brzuszkiewicz, Z Dauter, On the reproducibility of protein crystal structures: Five atomic resolution structures of trypsin. *Acta Crystallogr. Sect. D Biol. Crystallogr.* **69**, 1447–1462 (2013).
